# Real-time predictive model of extrauterine growth retardation in preterm infants with gestational age less than 32 weeks

**DOI:** 10.1038/s41598-024-63593-9

**Published:** 2024-06-05

**Authors:** Liang Gao, Wei Shen, Fan Wu, Jian Mao, Ling Liu, Yan-Mei Chang, Rong Zhang, Xiu-Zhen Ye, Yin-Ping Qiu, Li Ma, Rui Cheng, Hui Wu, Dong-Mei Chen, Ling Chen, Ping Xu, Hua Mei, San-Nan Wang, Fa-Lin Xu, Rong Ju, Zhi Zheng, Xin-Zhu Lin, Xiao-Mei Tong, The Chinese Multicenter EUGR Collaborative Group, Xinzhu Lin, Qianxin Tian, Yuan Yuan, Bizhen Shi, Xiao-Mei Tong, Jinghui Zhang, Yan Zhu, Xiuzhen Ye, Jingjing Zou, Yinping Qiu, Yuhuai Li, Shuhua Liu, Ying Xu, Wenli Zhou, Dongmei Chen, Zhiyong Liu, Sannan Wang, Falin Xu, Xiaokang Wang, Ye Liu, Juan Yi, Meigui Wu, Shifeng Chen, Qiaomian Zhu, Linlin Wang, Yongqiao Liu, Chun Deng, Xiaohong Liu

**Affiliations:** 1 Department of Neonatology, Women and Children’s Hospital, School of Medicine, Xiamen University, Xiamen, 361000 Fujian China; 2 Department of Neonatology, Third Affiliated Hospital of Guangzhou Medical University, Guangzhou, 510000 China; 3 Department of Pediatrics, Shengjing Hospital of China Medical University, Shenyang, 110000 China; 4 Department of Neonatology, Guiyang Maternal and Child Health Hospital and Guiyang Children’s Hospital, Guiyang, 550000 China; 5 Department of Pediatrics, Peking University Third Hospital, Beijing, 100000 China; 6 Department of Neonatology, Pediatric Hospital of Fudan University, Shanghai, 200001 China; 7 Department of Neonatology, Guangdong Province Maternal and Children’s Hospital, Guangzhou, 510000 China; 8 Department of Neonatology, General Hospital of Ningxia Medical University, Yinchuan, 750000 China; 9 Department of Neonatology, Children’s Hospital of Hebei Province, Shijiazhuang, 050000 China; 10 Department of Neonatology, Children’s Hospital of Nanjing Medical University, Nanjing, 210000 China; 11 Department of Neonatology, The First Hospital of Jilin University, Changchun, 130000 China; 12 Department of Neonatology, Quanzhou Maternity and Children’s Hospital, Fujian, 362000 Quanzhou China; 13 Department of Pediatrics, Tongji Hospital, Tongji Medical College, Huazhong University of Science and Technology, Wuhan, 430000 Hubei China; 14 Department of Neonatology, Liaocheng People’s Hospital, Liaocheng, 252000 Shandong China; 15 Department of Neonatology, The Affiliate Hospital of Inner Mongolia Medical University, Hohhot, 010010 Inner Mongolia China; 16 Department of Neonatology, Suzhou Municipal Hospital, Suzhou, 215002 Jiangsu China; 17 Department of Neonatology, The Third Affiliated Hospital of Zhengzhou University, Zhengzhou, 450052 Henan China; 18 Department of Neonatology, School of Medicine, Chengdu Women’ and Children’s Central Hospital, University of Electronic Science and Technology of China, Chengdu, 611731 Sichuan China; 19 Nutritional Committee of Neonatology Branch of Chinese Medical Doctor Association, National Multicenter EUGR Collaborative Group, Beijing, 100191 China; 20 Department of Neonatology, Shandong Provincial Hospital, Jinan, 250000 Shandong China; 21 Department of Neonatology, Shanghai Children’s Medical Center, Shanghai, 2000127 China; 22 Department of Neonatology, Hunan Children’s Hospital, Changsha, 410000 Hunan China; 23 Department of Neonatology, Guangzhou Women and Children’s Medical Center, Guangzhou, 510000 Guangdong China; 24 Department of Neonatology, The First People’s Hospital of Yulin, Yulin, 537099 Guangxi China; 25 Department of Neonatology, Xian Children’s Hospital, Xian, 710002 Shanxi China; 26 Department of Neonatology, Baoji Maternal and Child Health Hospital, Baoji, 721000 Shanxi China; 27 Department of Neonatology, People’s Hospital of Xinjiang Uygur Autonomous Region, Urumchi, 830000 Xinjiang China; 28 Department of Neonatology, Children’s Hospital of Chongqing Medical University, Chongqing, 400015 China; 29 Department of Pediatrics, Affiliated Hospital of Qingdao University, Qingdao, 266000 Shandong China

**Keywords:** Very preterm infant, Extrauterine growth retardation, Prediction, Nomogram, Medical research, Risk factors

## Abstract

The aim of this study was to develop a real-time risk prediction model for extrauterine growth retardation (EUGR). A total of 2514 very preterm infants were allocated into a training set and an external validation set. The most appropriate independent variables were screened using univariate analysis and Lasso regression with tenfold cross-validation, while the prediction model was designed using binary multivariate logistic regression. A visualization of the risk variables was created using a nomogram, while the calibration plot and receiver operating characteristic (ROC) curves were used to calibrate the prediction model. Clinical efficacy was assessed using the decision curve analysis (DCA) curves. Eight optimal predictors that namely birth weight, small for gestation age (SGA), hypertensive disease complicating pregnancy (HDCP), gestational diabetes mellitus (GDM), multiple births, cumulative duration of fasting, growth velocity and postnatal corticosteroids were introduced into the logistic regression equation to construct the EUGR prediction model. The area under the ROC curve of the training set and the external verification set was 83.1% and 84.6%, respectively. The calibration curve indicate that the model fits well. The DCA curve shows that the risk threshold for clinical application is 0–95% in both set. Introducing Birth weight, SGA, HDCP, GDM, Multiple births, Cumulative duration of fasting, Growth velocity and Postnatal corticosteroids into the nomogram increased its usefulness for predicting EUGR risk in very preterm infants.

## Introduction

The postnatal nutritional status of very preterm infants (VPI), and extrauterine growth retardation (EUGR) in particular is receiving increasing attention. Studies have shown that EUGR affects not only the physical development and immediate complications of very preterm infants, but also their long-term health, especially neurocognitive function, and may increase the risk of cardiovascular system diseases and chronic metabolic syndrome in adulthood^1^. EUGR is relative to intrauterine growth retardation(IUGR). However, until now there is still much controversy about the timing, population and criteria for the evaluation of EUGR. Data from a 2016 multicenter survey including 25,899 preterm infants with birth weight < 1500 g at 22–32 weeks of gestational age showed that the incidence of EUGR evaluated based on weight was 38%^2^. A multicenter cohort study in Shandong Province, China, in 2018 showed that the overall EUGR incidence was 60.7% in 1051 preterm infants with gestational age < 32 weeks or weight < 1500 g, compared with 53.7% in non-small for gestation age(SGA) preterm infants^3^. Figueras Aloy et al.^4^ followed up 479 very preterm infants less than 32 weeks of gestational age and the incidence of EUGR was 51% when they reached 34–36 weeks of gestational age. El Rafei et al.^5^ investigated 6792 preterm infants in 19 regions of 11 European countries and the incidence of EUGR remained at 60.3%. This indicates that EUGR is a common problem that very preterm infants need to face after birth. Exploring the risk factors of EUGR and early prediction of the risk of EUGR in VPI is currently a hot topic in neonatal medicine research. Low birth weight is considered the most important risk factor for EUGR by numerous studies^6,7^, and multiple pregnancies, hypertensive disease complicating pregnancy(HDCP), SGA and prenatal glucocorticoid use also increase the incidence of EUGR^5^. Many neonatal complications such as severe infections, neonatal respiratory distress syndrome(nRDS), bronchopulmonary dysplasia(BPD) and necrotizing enterocolitis(NEC)^8^, have also been shown to be risk factors for EUGR. Protein and energy are closely related to weight gain in the early postnatal period and increasing energy intake in the first postnatal week can reduce postnatal weight loss, while insufficient cumulative intake can directly lead to lagging weight gain in preterm infants, thus adversely affecting their growth and development^9^. The effect of gestational age on EUGR is controversial, with most studies suggesting that increasing gestational age reduces the risk of EUGR^10^, but some studies have found that increasing gestational age is an independent risk factor for EUGR^11^. EUGR is strongly associated with birth weight, gestational age, perinatal abnormalities, neonatal diseases, and inadequate nutritional support after birth. In fact, these risk factors have significant multicollinearity with each other, and most of the previous studies performed univariate analysis only at the screening stage of independent variables and analyzed the dominance ratio of risk factors by logistic regression equation, which does not deal well with the interference of various confounding factors. Moreover, when too many independent variables are included in the analysis, there is a high requirement for the sample size of the study cohort, and the results of studies with small sample sizes may suffer from insufficient test efficacy. Therefore, constructing a nomogram based on large-sample clinical data from multiple centers to provide a personalized, evidence-based, and highly accurate risk prediction model can predict the risk of EUGR in VPI at an early stage and facilitate decision-making related to clinical management, which is of great significance for improving the prognosis of very preterm infants and reducing the incidence of EUGR.

## Materials and methods

The present prediction model was written based on the TRIPOD list^12^.

### Study population

The study population was derived from a prospective multicenter study on extrauterine growth retardation in very preterm infants and the factors affecting it in 28 hospitals in level III NICUs in 7 regions of China from September 2019 to December 2020. The aim of original study was to investigate the incidence and related factors of extrauterine growth retardation in very premature infants during hospitalization from different regions of China. Therefore, although the original research design has fit this study in depth, strictly speaking, this study presented in the manuscript was a deep mining of the original data.

The study was registered in the Chinese Clinical Trials Registry (http://www.chictr.org.cn; registration number: ChiCTR1900023418). The study protocol was approved by the Ethics Committee of Women and Children's Hospital of Xiamen University/Xiamen Maternal and Child Health Hospital (Batch number: KY-2019-016), and all informed consent was obtained from the families of the children.

Inclusion criteria: 1. gestational age < 32 weeks; 2. hospitalization > 2 w; 3. admission within 24 h after birth. Exclusion criteria: 1. congenital structural malformations; 2. hospitalization less than 2 w; 3. inherited metabolic diseases; 4. death, interruption of treatment or automatic discharge during hospitalization; 5. incomplete data. Discharge criteria: 1. cure of primary disease and stable vital signs; 2. milk intake up to full enteral feeding; 3. weight 1800 ~ 2000 g or more.

### Sample size

This study was designed as a cohort study to investigate the prevalence of EUGR, based on the literature^13^, which reported that the prevalence of EUGR in preterm infants born at gestational age < 32 weeks ranged from 51 to 60%, setting the test level α at 0.05 and the permissible error δ at 2%. Thus, the required sample size was calculated to be 2305 cases, and the estimated missing data rate was 10%, so the total sample size was expected to be 2,500 cases.

### Data collection

The Women and Children's Hospital affiliated to Xiamen University was used as the survey center, and EpiData was applied to establish a database with a uniform format of questionnaire. The data of the case report form were entered in two-person duplex, and the data entry was performed strictly according to the requirements of the study protocol.

### Observation indicators

General indicators: The perinatal data of mothers were recorded, such as mode of delivery, antenatal corticosteroids (ACS) and gestational diabetes mellitus(GDM), HDCP, etc. The gestational age, birth weight, sex, postnatal glucocorticoid use, mechanical ventilation of preterm infants were also recorded.

Nutrition and developmental indicators: Nutritional and developmental indicators included time of initiation of enteral feeding, main milk feeding during hospitalization, time to reach total enteral feeding (150 ml/kg/d), cumulative days of fasting, cumulative time of parenteral nutrition, time to regain birth weight, growth velocity after regaining birthweight, cumulative calories in the first week after birth, cumulative amino acid and fat milk intake in the first week after birth, total calories up to 110 kcal/kg/d of age and oral calories up to 110 kcal/kg/d of age, etc.

Major complications in preterm infants: The major complications of prematurity include grade III-IV nRDS, moderate to severe BPD, stage II or higher NEC, grade III-IV intraventricular hemorrhage(IVH), periventricular leukomalacia (PVL), hemodynamically significant patent ductus arteriosus (hsPDA), early-onset sepsis (EOS), late-onset sepsis (LOS), retinopathy of prematurity requiring intervention (ROP), feeding intolerance (FI), metabolic bone disease of prematurity (MBPD), parenteral nutrition-associated cholestasis (PNAC), etc.

### Criteria for related diseases and treatment protocols

EUGR evaluation criteria: using the 2013 Fenton growth curve^14^ as a reference, correcting for weight, length and head circumference indicators below the 10th percentile of the growth curve at 36 weeks of gestational age or at hospital discharge.

SGA: refers to newborns whose birth weight is less than the 10th percentile of the mean birth weight for children of the same sex and gestational age.

Growth velocity (GV) (g/kg-d): the formula is GV^15^ = [1000 × ln(Wn/W1)]/(Dn-D1), where Wn is real-time weight (g), W1 is birth weight (g), Dn is the age in days (d), and D1 is time to regain birth weight (d). The body weight was measured every two days after birth, and growth velcoity was evaluated every week.

FI evaluation criteria: gastric residuals exceeding 50% of the previous feeding with vomiting and/or abdominal distention; feeding plan failure, including reduced, delayed or interrupted enteral feeding, with one of the two being diagnostic^16^.

Brain injury (BI) defined as the presence of grade III-IV IVH, PVL and hemorrhagic infarction on cranial ultrasound or cranial MRI.

Diagnostic criteria for MBDP: blood alkaline phosphatase > 900 IU/L with blood phosphorus < 1.8 mmol/L^17^.

Diagnostic criteria for PNAC: persistent PN ≥ 14 d, skin yellowing that cannot be explained by the original disease, hepatosplenomegaly, light-colored stools or white clay-like stools, serum conjugated bilirubin > 2 mg/dl or > 20% of total bilirubin, except for other biliary atresia, infections and cholestasis due to genetic metabolic diseases^18^.

NEC was defined as Bell stage ≥ 2, wherein the NEC suspected based on signs and symptoms was confirmed by pneumatosis intestinalis on radiology^19^.

A diagnosis of BPD was indicated by at least 28 days of oxygen therapy above the fraction of inspired oxygen (FiO_2_) of 0.21. Based on the FiO_2_ requirement at 36 weeks post-menstruation age or discharge, whichever came first, BPD was characterized as mild (room air), moderate (FiO_2_ < 0.3), or severe (FiO_2_ ≥ 0.3 or requiring positive pressure ventilation)^20^.

ROP that requires intervention: ROP requiring intravitreal drug injection, laser therapy, or surgery.

EOS and LOS include confirmed sepsis with positive blood cultures versus clinically diagnosed sepsis, with diagnostic criteria referring to the Expert Consensus on the Diagnosis and Treatment of Neonatal Sepsis (2019 edition)^21^.

hsPDA was considered when catheter diameter was > 1.5 mm, left atrial diameter/aortic diameter was ≥ 1.4 mm, and left-to-right shunt (or bi-directional bi-phase shunt) was needed. In addition, the PDA was accompanied by at least one of the following clinical manifestations: heart murmur, tachycardia, increased respiration, increased pulse pressure, hypotension, flushing or cardiac dilation.

nRDS grade III-IV, IVH grade III-IV, and PVL were diagnosed with reference to Practical Neonatology (5th edition)^22^.

### Statistical analysis

The data were processed for statistical analysis using R-software (version 4.1.3; R Foundation for Statistical Computing, Vienna, Austria). We have used the mice function of the mice package to perform multiple chain imputation on the original data based on the random forest rule, then we have got 5 data sets, and we have finally selected the data set obtained by the fourth imputation for statistical analysis. Continuous variables were expressed as median and interquartile spacing, and Kruskal–Wallis nonparametric test was used for comparison between groups. Count data were expressed as number of cases and percentages (%), and the Person chi-square test was used for comparison between groups. Differences were considered to be remarkable when P values below 0.05.

Stratified random sampling of the study cohort based on gestational age sorting across trial centers was performed using the sampling package in R software. The random seed was set at 666 in a ratio of 7:3 and divided into a training set of 1760 cases and an internal validation set of 754 cases.

In this study, the data of cases with missing outcome were directly deleted, and the absence of predictor variables was treated by multiple imputation using the mice package, the number of imputation was 5, including all the predictive factors and outcome variables in the imputation model; after the imputation, the analysis results of the imputation data were combined according to Rubin's rules.

After univariate analysis, LASSO regression with k-fold cross-validation (tenfold validation) was performed using the glmnet package in R software to select the independent variables for the prediction model, and the optimal lambda was selected as lambda.1se. At this time, 14 independent variables were obtained. Next, we eliminated 6 variables again according to the inverse stepwise selection based on the Akaike information criterion, and finally selected 8 optimal independent variables. Based on these 8 most suitable predictors, a prediction model was constructed by binary multi-factor logistic regression using the rms package, and a nomogram was drawn, and the risks of clinical characteristics were expressed by *OR* values, *P* values and 95% *CI*s.

Finally, the accuracy of the prediction model was estimated using the following validation methods in the training set and internal validation set, respectively. The *ROC* curves were plotted with the pROC package and the area under the curve was calculated to verify the discriminatory ability of the prediction model. Plotting Calibration curves with the rms package to assess the calibration of the prediction model. Plotting DCA benefit curves with the ggDCA package to evaluate the clinical efficacy of the prediction model.

### Ethics approval and consent to participate

The study was conducted in accordance with the Declaration of Helsinki, and approved by the Ethics Committee of Women and Children's Hospital affiliated to Xiamen University/Xiamen Maternal and Child Health Hospital (Batch number kY-2019–016). Consent to participate and for publication All participants gave written informed consent. The informed consent was obtained from the families of the children.

### Consent to participate and for publication

All participants gave written informed consent.

## Results

### Characteristic of the study cohort

**Figure 1:**
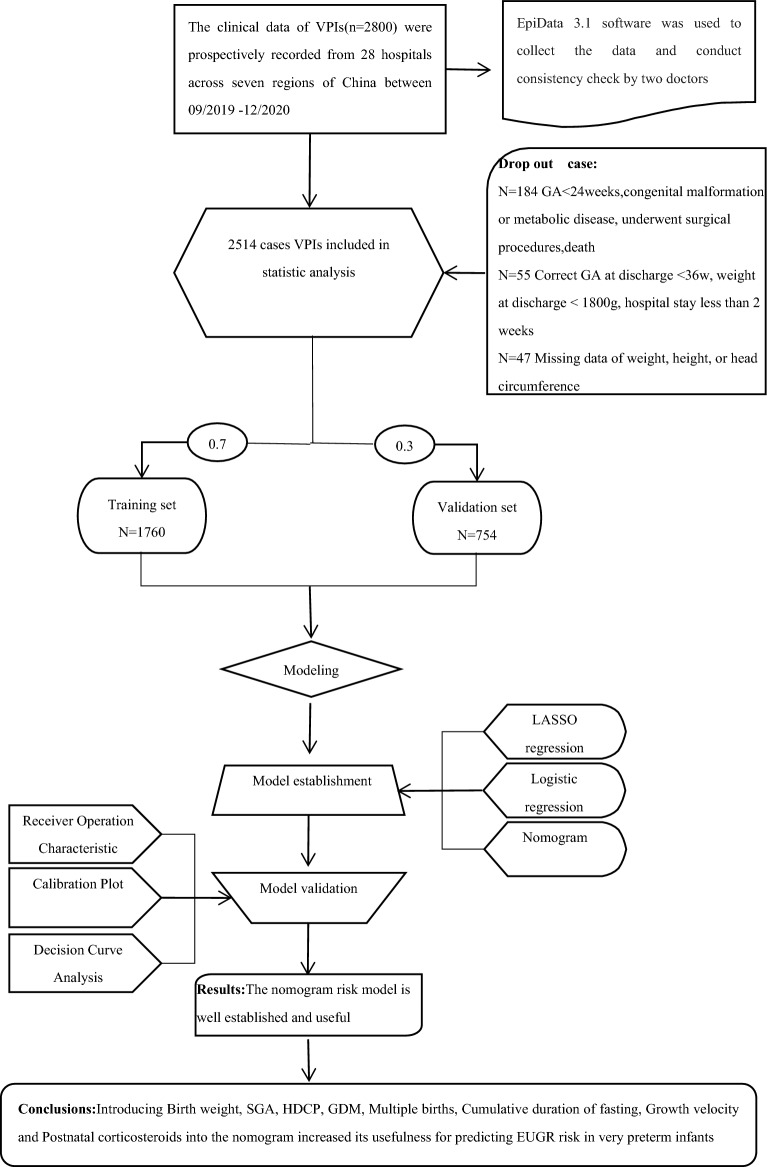
Flow chart of study design. *VPIs* very preterm infants, *GA* gestation age. Select the predictors and fit the model using only the training set.

Our study cohort included 2514 very preterm infants of gestational age 24–31^+6^ weeks. Based on weight assessment, 1189 preterm infants were combined with EUGR and 1325 non-EUGR preterm infants, with an incidence of EUGR of 47.3%. These preterm infants were randomly sampled in a 7:3 ratio according to gestational age into the training set and the validation set (7 in the training set and 3 in the validation set), with 1760 cases in the training set and 754 cases in the validation set, as shown in Fig. 1. The basic characteristics of the two groups of preterm infants were shown in Table 1. There were not statistically significant differences in the independent variables between the training set and the validation set. So, the training set and the validation set obtained were well representative of the overall population.

**Table 1:** Characteristics of the 2514 preterm infants with gestational age less than 32 weeks.

Variables		Total cohort (n = 2514)	Infants with EUGR (n = 1189)	Infants without EUGR (n = 1325)	*P* value	Training set (n = 1760)	Validation set (n = 754)
Gestation age, w		30.1 (28.9, 31.1)	30 (28.7, 31.0)	30.3 (29.0, 31.1)	0.001	30.1 (28.9, 31.1)	30.3 (28.9, 31.1)
Birth weight, g		1331 (1130, 1540)	1200 (1009, 1370)	1470 (1280, 1650)	< 0.001	1340 (1130, 1540)	1320 (1100, 1530)
Apgar 5-min		9 (8, 9)	9 (8, 9)	9 (8, 10)	< 0.001	9 (8, 9)	9 (8, 9)
Initiation of enteral nutrition, h		23 (8, 40)	24 (12.0, 48.0)	20 (6.0, 30.0)	< 0.001	22 (8, 38.9)	24 (10, 46)
Milk volume at initiation of HMF, ml		0 (0, 100)	0 (0, 100)	0 (0, 98.5)	0.778	0 (0, 100)	0 (0, 100)
Duration of fasting, d		2 (0.8, 5.0)	3.0 (1.0, 7.0)	1.0 (0.4, 3.0)	< 0.001	2 (0.8, 5.0)	2 (0.8, 5.0)
Days of achieving total enteral feeding, d		25 (17, 36)	30 (21, 40)	21 (15, 31)	< 0.001	25 (17, 35)	25 (17, 36)
Duration of PN, d		21 (13, 31)	24 (17, 35)	28 (11, 26)	< 0.001	21 (14, 30)	21 (13, 32)
The cumulative total amino acids intake in the first week, g		16 (13.3, 18.5)	16.3 (13.8, 18.6)	16 (12.9, 18.2)	< 0.001	16.1 (13.4, 18.5)	16 (13.3, 18.2)
The cumulative total amino acids intake in hospital, g		44.8 (26.4, 72.5)	52.4 (35.0, 83.4)	37.9 (21.0, 59.1)	< 0.001	45.0 (26.5, 72.0)	44.5 (26.0, 75.0)
The cumulative total fat intake in the first week, g		12.6 (7.5, 15.0)	13.0 (10.5, 15.0)	12.2 (9.4, 14.8)	< 0.001	12.7 (10, 15)	12.4 (10, 15)
The cumulative total fat intake in hospital, g		37.7 (21.0, 60.7)	44.7 (28.5, 71.9)	31.8 (16.5, 51.0)	< 0.001	37.5 (21, 59.7)	38 (20.7, 62.3)
The cumulative caloric intake in the first week, Kcal		496.4 (421.0, 563.9)	410.7 (355.0, 480.0)	512.4 (433.0, 579.2)	< 0.001	496.2 (419.6, 565.6)	496.8 (424.2, 562.1)
Days of total caloric intake up to 110 kcal/kg/d, d		9 (7, 14)	10 (7, 17)	8 (6, 12)	< 0.001	9 (7, 15)	9 (7, 14)
Days of oral administration up to 110 kcal/kg/d, d		23 (16, 33)	28 (20, 39)	20 (14, 29)	< 0.001	23 (16, 33)	23 (16, 34)
Percentage of physiological weight loss, %		6.3 (3.6, 9.2)	6.3 (3.5, 9.5)	6.3 (3.7, 9.0)	0.643	6.3 (3.5, 9.2)	6.3 (3.6, 9.2)
Days of birth weight recovery, d		9 (7, 12)	9 (7, 12)	9 (7, 11.5)	0.127	9 (7, 12)	9 (7, 12)
Growth velocity, g/kg/d		15.1 (12.8, 18.2)	14.3 (12.0, 17.2)	15.9 (13.5, 19.3)	< 0.001	15.0 (12..7, 18.2)	15.2 (12.8, 18.1)
Duratoin of hospital stay, d		46 (35, 60)	53 (42, 66)	39 (31, 51)	< 0.001	46 (35, 60)	46 (35, 60)
Duration of IMV, d		0 (0, 3)	1 (0, 6)	0 (0, 2)	< 0.001	0 (0, 3)	0.4 (0, 3.4)
Duration of NIMV, d		14.9 (6.0, 28.0)	19 (8, 31)	11 (5, 24)	< 0.001	14 (6, 28)	15 (6, 27)
Duration of oxygen therapy, d		8.0 (2.0, 17.9)	10 (3, 19)	7 (1, 16)	< 0.001	8 (2, 18)	8 (2, 17)
Duration of antibiotic therapy, d		11 (7, 20)	14 (7, 22)	10 (6, 17)	< 0.001	11 (7, 19)	12 (7, 20)
Number of blood transfusion		1 (0, 2)	2 (0, 3)	0 (0, 2)	< 0.001	1 (0, 2)	1 (0, 2)
Sex-Male		1378 (54.9%)	642 (54.0%)	736 (55.6%)	0.355	977 (55.5%)	401 (53.3%)
Cesarean delivery		1539 (61.2%)	807 (68.8%)	732 (55.3)	< 0.001	1055 (59.5%)	481 (63.8%)
HDCP		517 (20.6%)	363 (30.5%)	154 (11.6%)	< 0.001	348 (19.8%)	170 (22.5%)
GDM		437 (17.4%)	174 (14.6%)	263 (19.9%)	0.001	299 (17.0%)	138 (11.6%)
Multi births	1	1694 (67.4%)	781 (65.7%)	913 (68.9%)	0.014	1201 (68.2%)	493(65.4%)
	2	754 (30.0%)	366 (30.8%)	388 (29.3%)		515 (29.3%)	239(31.7%)
	3	66 (2.6%)	42 (3.5%)	24 (1.8%)		44 (2.5%)	22(2.9%)
ACS	0	556 (22.1%)	286 (24.1%)	270 (20.4%)	0.02	392 (22.3%)	164(21.8%)
	1	734 (29.2%)	322 (27.1%)	412 (31.1%)		507 (28.8%)	227(30.1%)
	2	1224 (48.7%)	581 (48.9%)	643 (48.5%)		861 (48.9%)	363(48.1%)
SGA		133 (5.3%)	131 (11.0%)	2 (0.2%)	0.000	90 (5.1%)	43 (5.7%)
Breast milk for starting feeds		362 (14.4%)	158 (13.3%)	204 (15.4%)	0.133	247 (14.0%)	115 (15.3%)
Breast-feeding		1075 (42.8%)	511 (43.0%)	564 (42.6%)	0.835	761 (43.2%)	314 (41.6%)
Central venous catheter		458 (18.2%)	126 (10.6%)	332 (25.1%)	0.000	312 (17.7%)	146 (19.4%)
Use of antibiotics		2399 (95.4%)	1148 (96.6%)	1251 (94.4%)	0.010	1674 (95.1%)	725 (96.2%)
hsPDA		431 (17.1%)	264 (22.2%)	167 (12.6%)	0.000	295 (16.8%)	136 (18.0%)
ROP-rec		80 (3.2%)	51 (4.3%)	29 (2.2%)	0.003	57 (3.2%)	23 (3.1%)
NEC(> 2)		212 (8.4%)	137 (11.6%)	75 (5.6%)	0.000	144 (8.2%)	68 (9.0%)
RDS(2–3)		384 (15.3%)	223 (18.8%)	161 (12.2%)	0.000	265 (11.1%)	119 (15.8%)
BPD(> m)		407 (16.2%)	264 (22.2%)	143 (10.8%)	0.000	279 (15.9%)	128 (17.0%)
Postnatal corticosteroids		353 (14.0%)	223 (18.8%)	130 (9.8%)	0.000	245 (13.9%)	108 (14.3%)
BI		187 (7.4%)	92 (7.7%)	95 (7.2%)	0.588	133 (7.6%)	54 (7.2%)
EOS		369 (14.7%)	182 (15.3%)	187 (14.1%)	0.398	270 (15.3%)	99 (13.1%)
LOS		327 (13.0%)	201 (16.9%)	126 (9.5%)	0.000	223 (12.7%)	104 (13.8%)
FI		917 (36.5%)	522 (43.9%)	395 (29.8%)	0.000	641 (36.4%)	276 (36.6%)
MBDP		73 (2.9%)	45 (3.8%)	28 (2.1%)	0.013	48 (2.7%)	25 (3.3%)
PNAC		263 (10.5%)	175 (14.8%)	87 (6.6%)	0.000	187 (10.7%)	75 (9.9%)

Continuous variables are displayed as medians (25% quartile, 75% quartile). Categorical variables are displayed as numbers and percentages.

*EUGR* extrauterine growth retardation, *SGA* small for gestation age, *HDCP* hypertensive disease complicating pregnancy, *GDM* gestational diabetes mellitus, *BI* brain injury, *EOS* early-onset sepsis, *LOS* late-onset sepsis, MBDP metabolic bone disease of prematurity, *PNAC* parenteral nutrition-associated cholestasis, *BPD* moderate to severe bronchopulmonary dysplasia, *nRDS* III–IV neonatal respiratory distress syndrome, *NEC* stage II or higher necrotizing enterocolitis, *ROP* ROP requiring intravitreal drug injection, laser therapy, or surgery, *IMV* Invasive mechanical ventilation, *NIMV* non-invasive mechanical ventilation, *ACS* antenatal corticosteroids, *PDA* hemodynamically significant patent ductus arteriosus, *HMF* human milk fortifier, *PN* parenteral nutrition, *IQR* inter quartile range.

### Independent risk factors in the training set

**Figure 2:**
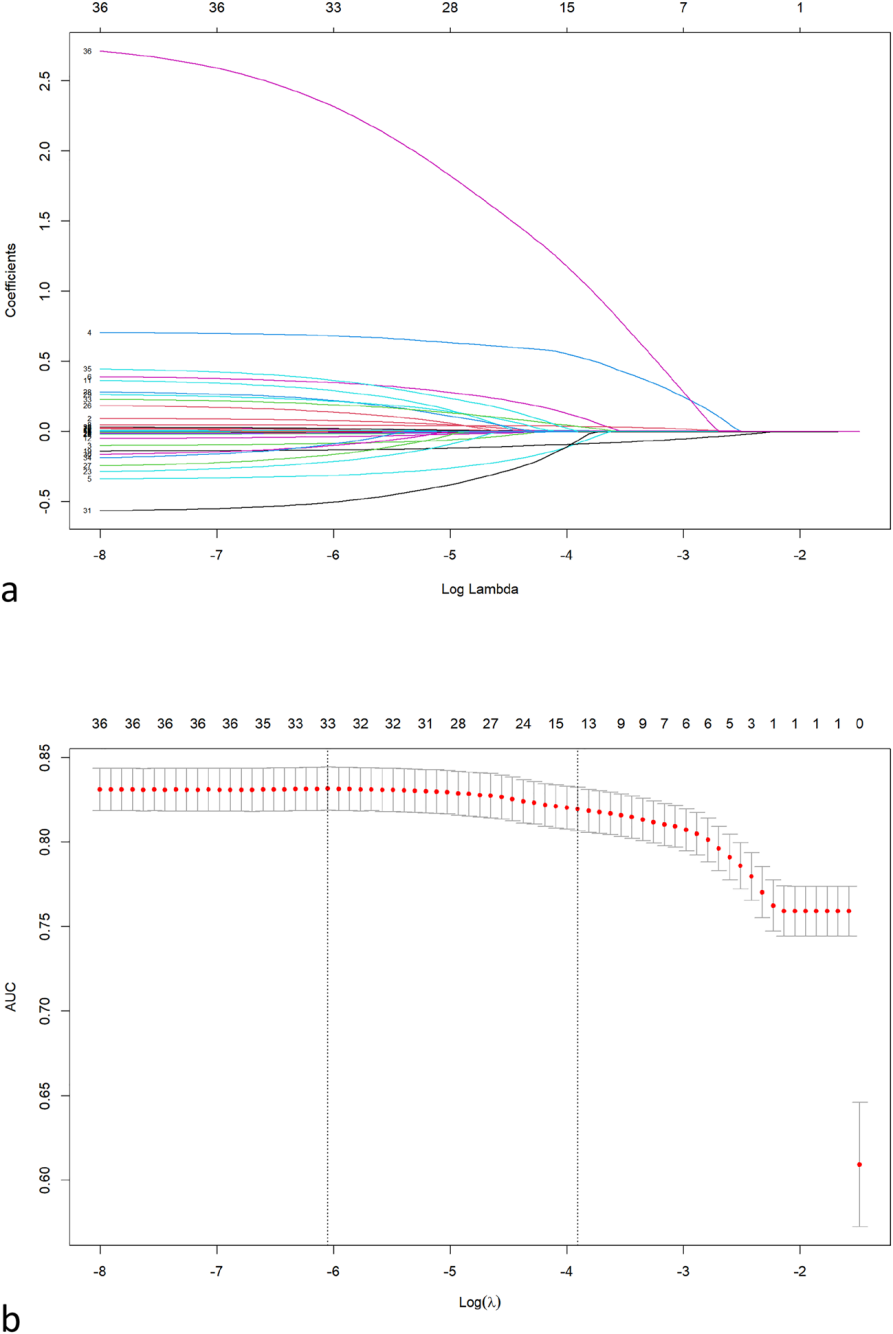
Variable selection by the LASSO regression analysis with k-fold cross-validation in the training set. (**a**) Forteen variables with nonzero coeffificients were selected by deriving the optimal lambda. (**b**) we plotted the AUC curve versus log(lambda) and drew dotted vertical lines based on 1 standard error criteria.

Univariate analysis revealed differences in 37 of the 49 clinical indicators. Predictor variables were selected from these variables by LASSO regression with k-fold cross-validation, and 14 predictor variables were screened, as shown in Fig. 2. Then, the inverse stepwise selection based on the Akaike information criterion resulted in the inclusion of 8 best-fit predictors, namely birth weight, SGA, HDCP, GDM, multiple births, cumulative duration of fasting, growth velocity and postnatal corticosteroids. The AIC values corresponding to the full model, the model fitted with 14 independent variables and the model fitted with 8 independent variables were 1393.8, 1770.5 and 1776.7, respectively.

### Predictive model construction

**Figure 3:**
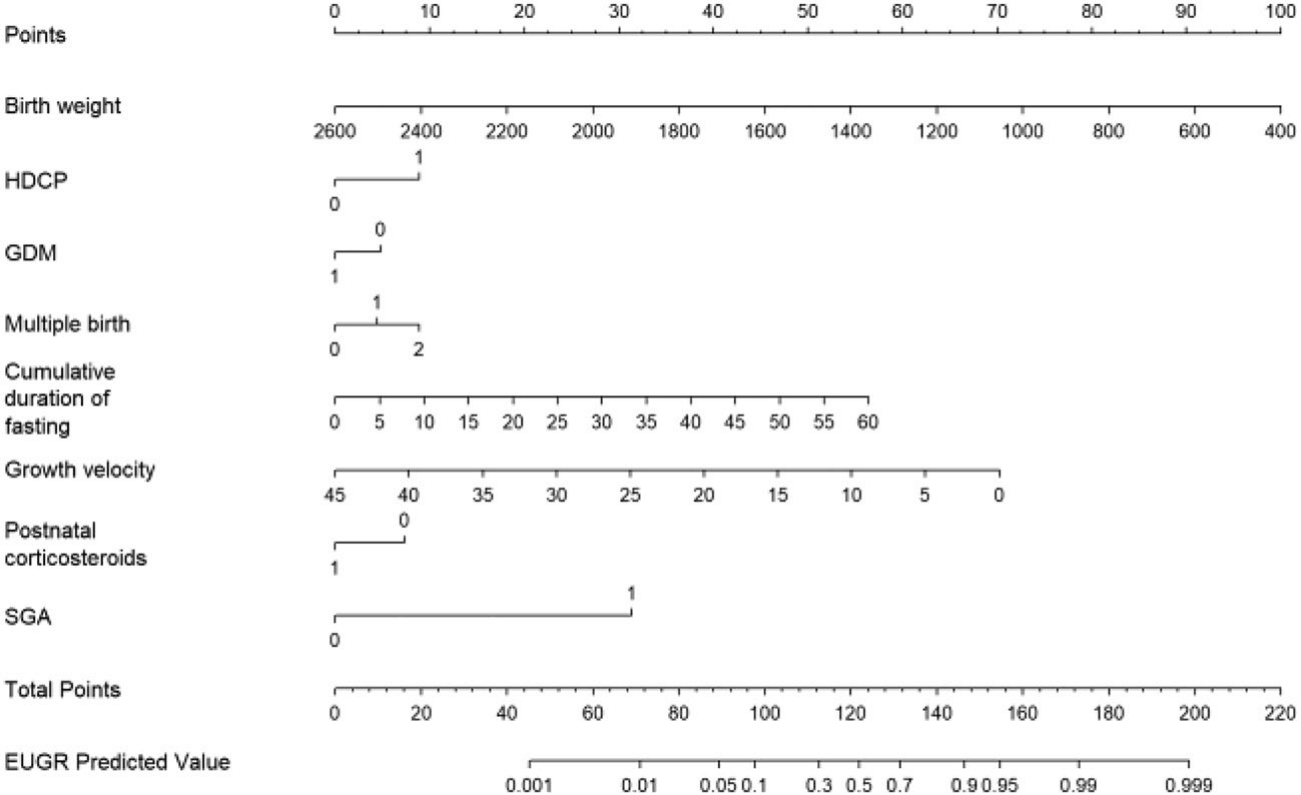
Risk factors of birth weight, SGA, HDCP, GDM, multiple births, cumulative duration of fasting, growth velocity and postnatal corticosteroids for nomogram prediction model. *EUGR* extrauterine growth retardation, *SGA* small for gestation age, *HDCP* hypertensive disease complicating pregnancy, *GDM* gestational diabetes mellitus.

Eight of the initial 49 variables were included in the predictive model. Since all eight predictors showed statistically significant differences (shown in Table 2) and were independent of each other(all variance inflation factors were less than 2), they were all introduced into the predictive model and a nomogram of EUGR risk probabilities was constructed (shown in Fig. 3). The endpoints of each variable were vertically upward to the score scale in the first column to obtain the corresponding score, and the scores of the eight variables were summed to obtain the total score, which was used to determine the risk probability of EUGR for patients. For example: a preterm infant with birth weight 1000 g, singleton, SGA, cumulative fasting period of 10 days, so the baby will have total points of 132, and EUGR predictive value of 70%. In addition, when the total score is below 40, the EUGR risk of preterm infants is extremely low, and we believe that EGUR prediction is unnecessary at this time.

**Table 2:** Logistic regression analysis of the predictors for the risk of EUGR in the training set.

Intercept and variables		Prediction model		
		Odds ratio	Confidence interval (2.5%)	Confidence interval (97.5%)
Intercept		1219	461.5	3221
Birth weight		0.996	0.995	0.996
HDCP		2.227	1.645	3.012
Multiple births	1			
	2	1.363	1.058	1.755
	3	3.516	1.619	7.636
GDM		0.650	0.477	0.884
SGA		16.609	3.902	70.689
Growth velocity		0.868	0.845	0.891
Cumulative duration of fasting		1.089	1.059	1.121
Postnatal corticosteroids		0.516	0.364	0.731

*EUGR* extrauterine growth retardation, *SGA* small for gestation age, *HDCP* hypertensive disease complicating pregnancy, *GDM* gestational diabetes mellitus.

### Predictive model validation

**Figure 4:**
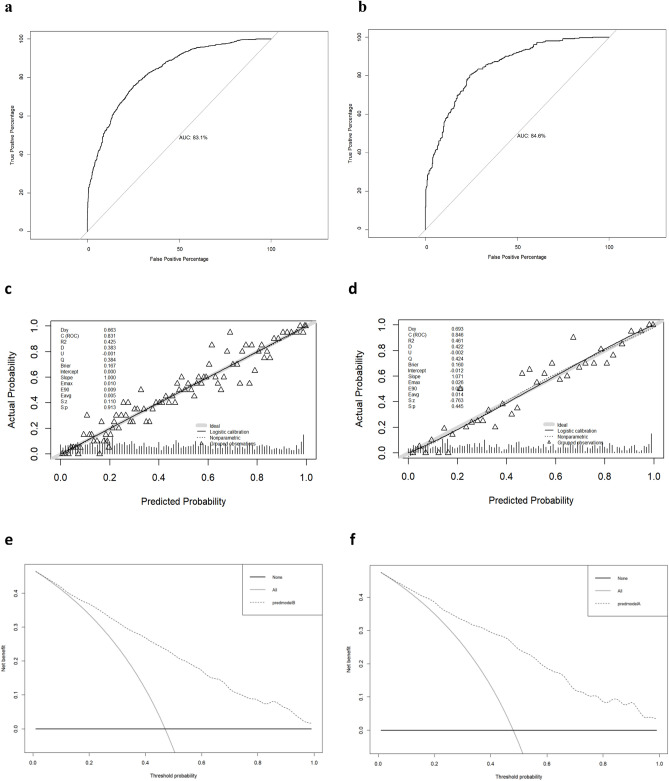
Validation of the EUGR risk nomogram. Receiver operating characteristic curve validation of the EUGR risk nomogram in the training set (**a**) and validation set (**b**). Calibration curves of the predictive EUGR risk nomogram in the training set (**c**) and validation set (**d**). Decision curve analysis for the EUGR risk nomogram in the training set (**e**) and validation set (**f**). *EUGR* extrauterine growth retardation.

The ROC curve was used to evaluate the discriminatory ability of the predictive model. The AUC of this predictive model was 83.1% in the training set and 84.6% in the validation set (shown in Fig. 4a,b), suggesting that the model has good discrimination. The AUC in the validation set was higher, which may be due to the following reasons: The study cohort was divided into 1760 training sets and 754 internal validation sets by stratified random sampling based on gestational age ranking in different trial centers. The baby of the training set and validation set were from the same population, and there was little difference in data between two group; 2. The sample size of the validation set is small; 3. The proportion of low-risk population in the validation set may be higher.

The prediction model was calibrated using calibration plots and the Hosmer–lemeshow test. The calibration curves, as well as the Hosmer–lemeshow test, showed that the predicted probabilities were highly consistent with the actual probabilities (*P* = 0.913 for the training set; *P* = 0.445 for the validation set) (shown in Fig. 4c,d), and the prediction model showed a good degree of fit in both the training and validation sets. The DCA curves show that the prediction model achieves good net benefit within a wide threshold of 0–95% in both the training and validation sets (shown in Fig. 4e,f).

## Discussion

The nomogram is a graphical visualization of the interrelationship between variables for outcome prediction based on the results of multifactorial analysis, integrating multiple predictors and assigning them according to certain proportions. Currently, there are no reports on the application of nomogram line graphs in EUGR management. To our knowledge, this is the first study to construct a nomogram for risk assessment of EUGR in preterm infants born at less than 32 weeks of gestational age.

EUGR, an abnormal disease state commonly found in very preterm infants, which is the result of a combination of influencing factors. In this process, various confounding factors must be considered as variables and the issue of multicollinearity among different variables. In this prospective study, we designed a more comprehensive list of factors and used LASSO regression analysis with k-fold cross-validation to screen variables and then divided the study cohort in a 7:3 ratio. The accuracy and stability of the model were modeled by the training set and validated by the internal validation set, and thus the method we chose could better address the accuracy and stability of the model. The probability of risk of EUGR in preterm infants born at gestational age < 32 weeks was well predicted by our prediction model constructed using eight factors, including birth weight,SGA, HDCP, GDM, multiple births, cumulative duration of fasting, growth velocity and postnatal corticosteroids. Although most of the factors were similar to those reported in previous studies, the statistical methods of this study differed significantly from these previous studies, providing more accurate results while demonstrating the contribution of each predictor to the risk probability of EUGR.

Birth weight is a key determinant of the occurrence of EUGR. The occurrence of many diseases is negatively correlated with birth weight, and as birth weight increases, the incidence of these diseases progressively decreases, and the corresponding medical interventions are reduced^23,24^. Thus, lower birth weight preterm infants are at higher risk for disease and take longer to achieve catch-up growth, making low birth weight preterm infants more likely to deviate from the growth curve and result in EUGR.

SGA is the adverse outcome of IUGR of the fetus itself. The cumulative retardation of intrauterine development will directly affect the growth and development of the fetus after birth^4^. The secondary EUGR is the continuation of intrauterine growth retardation. Controlled for other confounding factors in their study, Lima et al.^25^ found that SGA was the most influential variable for the weight (PR = 4.33) and head circumference (PR = 2.11) of preterm infants.The occurrence of IUGR is related to maternal obstetric diseases and nutritional status during pregnancy, such as HDCP. The basic pathophysiological changes of HDCP are parturient systemic small vessel spasm and increased peripheral vascular resistance, leading to reduced utero-placental blood supply and decreased placental function, which are more obvious when combined with adverse factors such as preeclampsia or decreased umbilical artery blood flow during diastolic period, thus causing fetal intrauterine growth retardation and intrauterine distress^26^. Even if the fetus was not evaluated as SGA at birth, its birth weight relative to gestational age was still small, which was closer to P10 on Fenton's intrauterine growth curve. Radmacherc et al. found that p-value of birth weight was an important predictor of EUGR, and a lower P-value was associated with an increased risk of EUGR^6^. In addition, intrauterine fetal gastrointestinal ischemia caused by HDCP increases the risk of postnatal feeding problems and even NEC in preterm infants, and affects their ability to digest and absorb food and establish enteral feeding, which may also increase the risk of EUGR^27^.

Fetal overgrowth is a common complication of GDM, and the risk of macrosomia in pregnant women with GDM may be about 3 times that in pregnant women with normal glucose tolerance^28^. Maternal hyperglycemia can lead to fetal hyperinsulinemia and an increase in unused glucose, which promotes adipose tissue and protein synthesis, resulting in fetal overgrowth in utero. The advantage of intrauterine growth and development may make it maintain good growth or evaluation advantage for a long time after birth. The slight deviation of growth curve in very preterm infants may partially compensate for this, thereby reducing the incidence of EUGR. However, birth weight increase is not always a benign event. Pregnant women with GDM are often associated with an increased risk of perinatal adverse outcomes such as prolonged labor, dystocia, fetal malformation, congenital heart disease, neonatal respiratory distress syndrome, and neonatal hypoglycemia^29^. It also increases the risk of obesity, diabetes and other metabolic diseases in adulthood^30^.

Multiple pregnancies increase many perinatal complications, including fetal anomalies, pre-eclampsia and gestational diabetes mellitus. Multiple pregnancies are associated with an increased risk of fetal and infant morbidity and mortality due to preterm birth and complications associated with prematurity as a result of multiple pregnancies^31,32^. This effect is significantly and positively correlated with the number of fetuses. Even when gestational age at birth is taken into account, infants with multiple pregnancies face more preterm complications than singleton infants born at the same gestational age^33^.

The growth rate of preterm infants during their NICU stay has a significant independent effect on neurological and physical growth outcomes. Several previous studies have concluded that the rate of neonatal weight gain at 24–38 weeks of gestational age is approximately 17 g/kg/d^14,34^ and that the rate of postnatal weight gain is closely related to nutritional management and depends on the accumulated protein, caloric and fluid intake in the early postnatal period^13^. Good nutritional management can meet the needs of preterm infants to achieve intrauterine growth rate and thus reduce the risk of EUGR.

Aggressive enteral nutrition is the optimal strategy for nutritional management of preterm infants^35^, with specific measures including early initiation of enteral nutrition, breastfeeding, application of breast milk fortification^36,37^, and avoidance of unnecessary milk reduction and fasting. The main causes of repeated multiple fasting in preterm infants are feeding intolerance and NEC, and prolonged fasting can cause gastrointestinal mucosal atrophy and peristaltic dysfunction, which are important influencing factors for the successful establishment of enteral feeding. Feeding intolerance and prolonged fasting inevitably delay the time to achieve total enteral feeding in preterm infants and increase the need for intravenous nutrition, thereby increasing the risk of complications associated with parenteral nutrition.

Glucocorticoids can reduce lung tissue edema by inhibiting lung inflammation, promote the generation of lung antioxidant enzymes and pulmonary surfactant, help shorten the time of ventilator adjuvant therapy, and reduce the incidence of BPD. Low dose, short course of systemic dexamethasone has been widely used in premature infants who rely on mechanical ventilation for a long time^38,39^. Systemic glucocorticoid therapy can improve the success rate of ventilator withdrawal and reduce the incidence of BPD in preterm infants who are still dependent on mechanical ventilation and have high risk factors for BPD at 7–14 days after birth^40^. Poets et al.^41^ also suggested that systemic dexamethasone treatment for ultra-low birth weight infants who were still dependent on mechanical ventilation and oxygen 14 days after birth could help to avoid BPD. The mechanisms of EUGR in children with BPD include insufficient nutritional support, increased respiratory work and resting metabolic rate, intermittent hypoxia, restricted fluid intake, use of diuretics and glucocorticoids, frequent pulmonary infection or sepsis, and feeding difficulties, which aggravate the undernutrition status of preterm infants and increase the risk of EUGR^42^. The use of postnatal glucocorticoids was positively correlated with the incidence of BPD. Although univariate analysis showed that the use of glucocorticoids was significantly higher in children with EUGR, glucocorticoids still played a positive role in preventing the occurrence of EUGR by reducing the incidence of BPD and other complications.

However, there are several limitations to our study. First, due to regional differences and heterogeneity in clinical practice among centers, multi center effects cannot be completely avoided, so there is a possibility of bias in the study results. Second, the study was conducted during the spread of the CoVID-19 epidemic, and the provision of breast milk was restricted due to epidemic prevention policies, resulting in a low breastfeeding rate in this study cohort.

## Conclusion

In conclusion, based on 8 factors including birth weight, SGA, HDCP, GDM, multiple births, cumulative duration of fasting, growth velocity and postnatal corticosteroids, we constructed the first predictive model of EUGR risk in very preterm infants. Internal validation confirmed good accuracy, consistency, and broad net benefit of the model. The visualized predictive model demonstrates the risk weights of different predictors, providing clinicians with a simple, intuitive and practical predictive tool that can provide better individualized predictive risk assessment for choosing the most optimal treatment strategy for preterm infants born after gestational age < 32 w.

### Supplementary Information

Supplementary Information.

## Data Availability

The datasets used during the current study are available from the corresponding author upon reasonable request.
