# Supplementary material for: Real-time predictive model of extrauterine growth retardation in preterm infants with gestational age less than 32 weeks
Source: Sci Rep. 2024 Jun 5;14:12884. doi: 10.1038/s41598-024-63593-9 (PMC11153599; doi:10.1038/s41598-024-63593-9)
Supplement: Supplementary file 1 — Supplementary Information. [file 41598_2024_63593_MOESM1_ESM.docx]

**Supplement figures and tables**

**Supplement table1: Logistic regression analysis of the predictors for the risk of EUGR in the training set.**

| **Intercept and Variables** | | **Prediction model** | | |
| --- | --- | --- | --- | --- |
|  |  | **Odds ratio** | **Confidence interval (2.5%)** | **Confidence interval (97.5%)** |
| Intercept | | 1033.11 | 282.43 | 3918.64 |
| Birth weight | | 0.9961 | 0.9955 | 0.9966 |
| HDCP | | 2.2536 | 1.6642 | 3.0638 |
| Multiple births | 1 | ref | ref | ref |
|  | 2 | 1.3574 | 1.0526 | 1.7518 |
|  | 3 | 3.9381 | 1.8358 | 8.6976 |
| GDM | | 0.6576 | 0.4810 | 0.8955 |
| SGA | | 16.3567 | 4.8330 | 102.5047 |
| Growth Velocity | | 0.8754 | 0.8513 | 0.8992 |
| Cumulative duration of fasting | | 1.0566 | 1.0237 | 1.0916 |
| Postnatal corticosteroids | | 0.5278 | 0.3706 | 0.7494 |
| Feeding intolerance | | 1.1744 | 0.9094 | 1.5157 |
| Cumulative caloric intake in the first week | | 0.9989 | 0.9978 | 1.0001 |
| Days of total caloric intake up to 110kcal/kg/d | | 1.0095 | 0.9918 | 1.0280 |
| Days of caloric up to 110kcal/kg/d | | 1.0044 | 0.9937 | 1.0154 |
| PNAC | | 1.3860 | 0.9225 | 2.0958 |


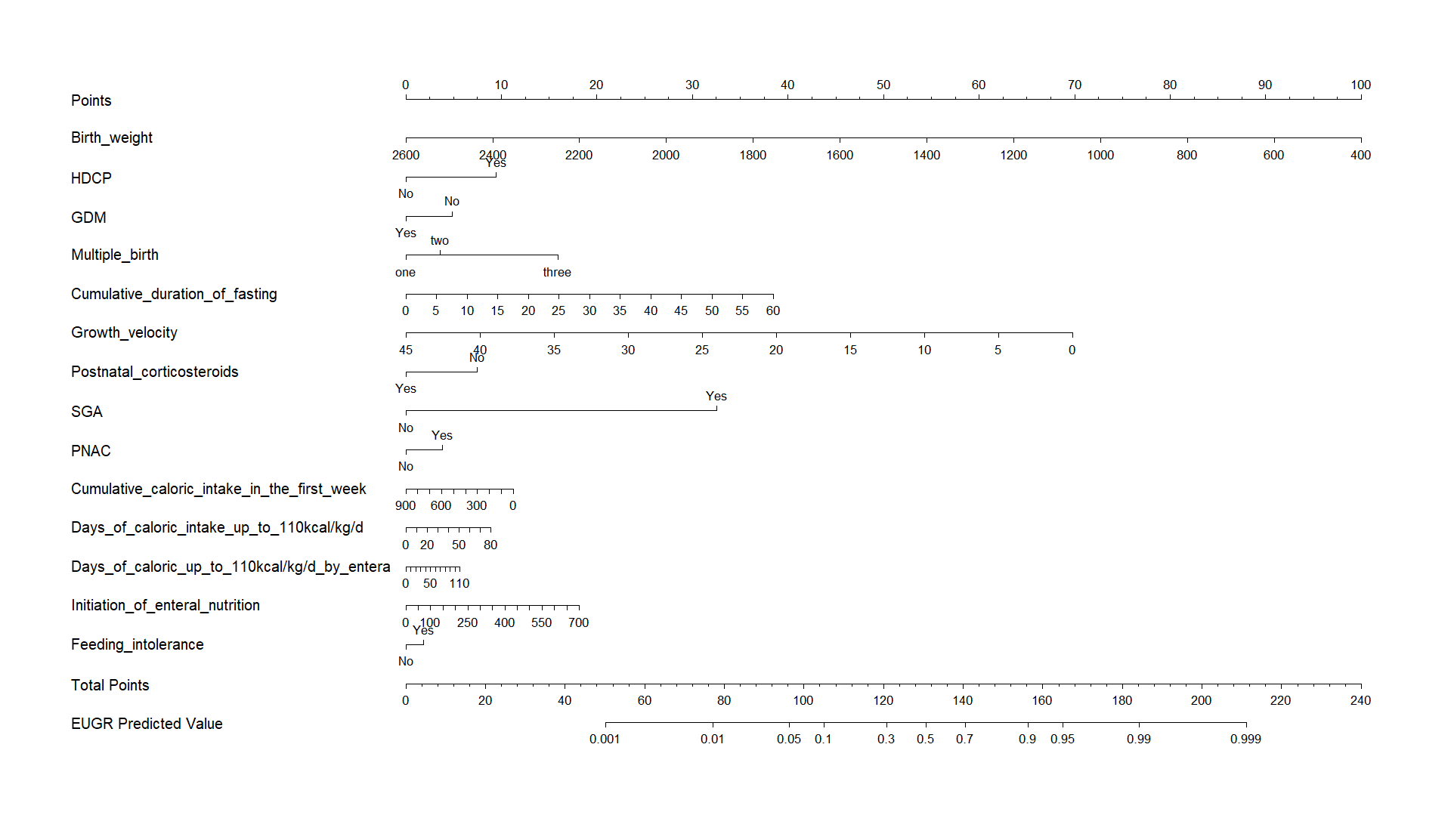


**Supplement figure1. Risk factors of birth weight, SGA, HDCP, GDM, multiple births, cumulative duration of fasting, growth velocity, postnatal corticosteroids, PNAC, feeding intolerance, cumulative caloric intake in the first week, days of total caloric intake up to 110kcal/kg/d, and days of caloric up to 110kcal/kg/d by entera for nomogram prediction model**


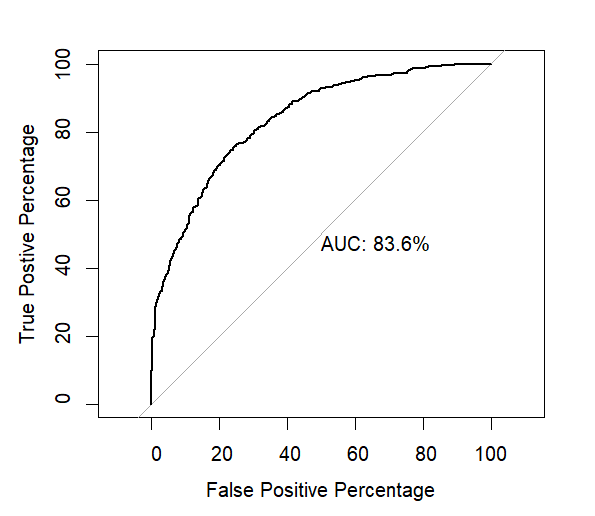


**Supplement figure 2. Receiver operating characteristic curve validation of the EUGR risk nomogram in the training set**


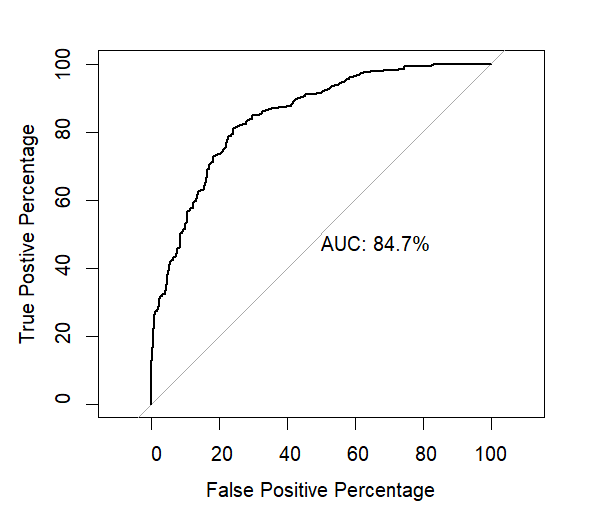


**Supplement figure 3. Receiver operating characteristic curve validation of the EUGR risk nomogram in validation set**


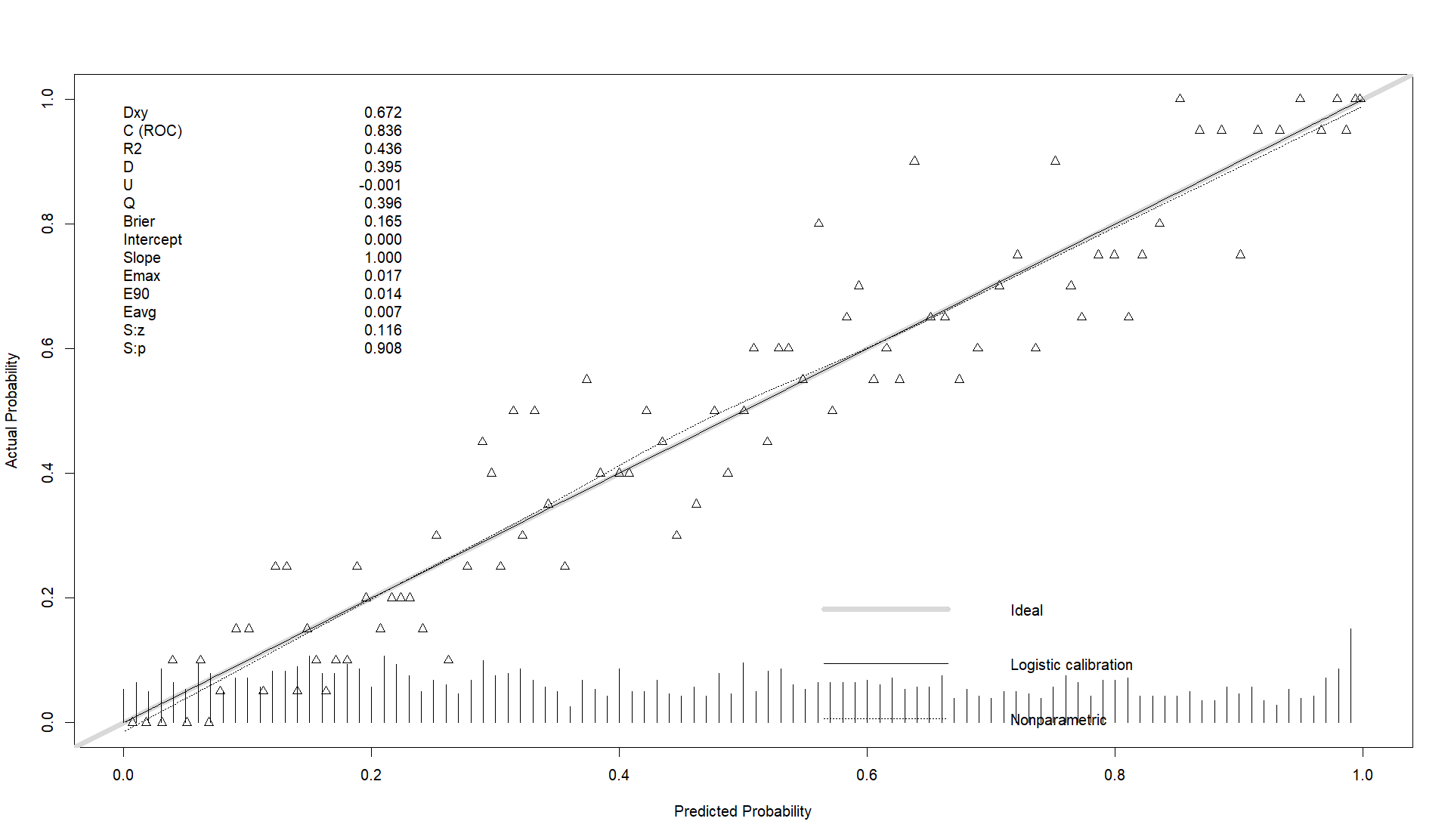


**Supplement figure 4. Calibration** **curves of the predictive EUGR risk nomogram in the training set**


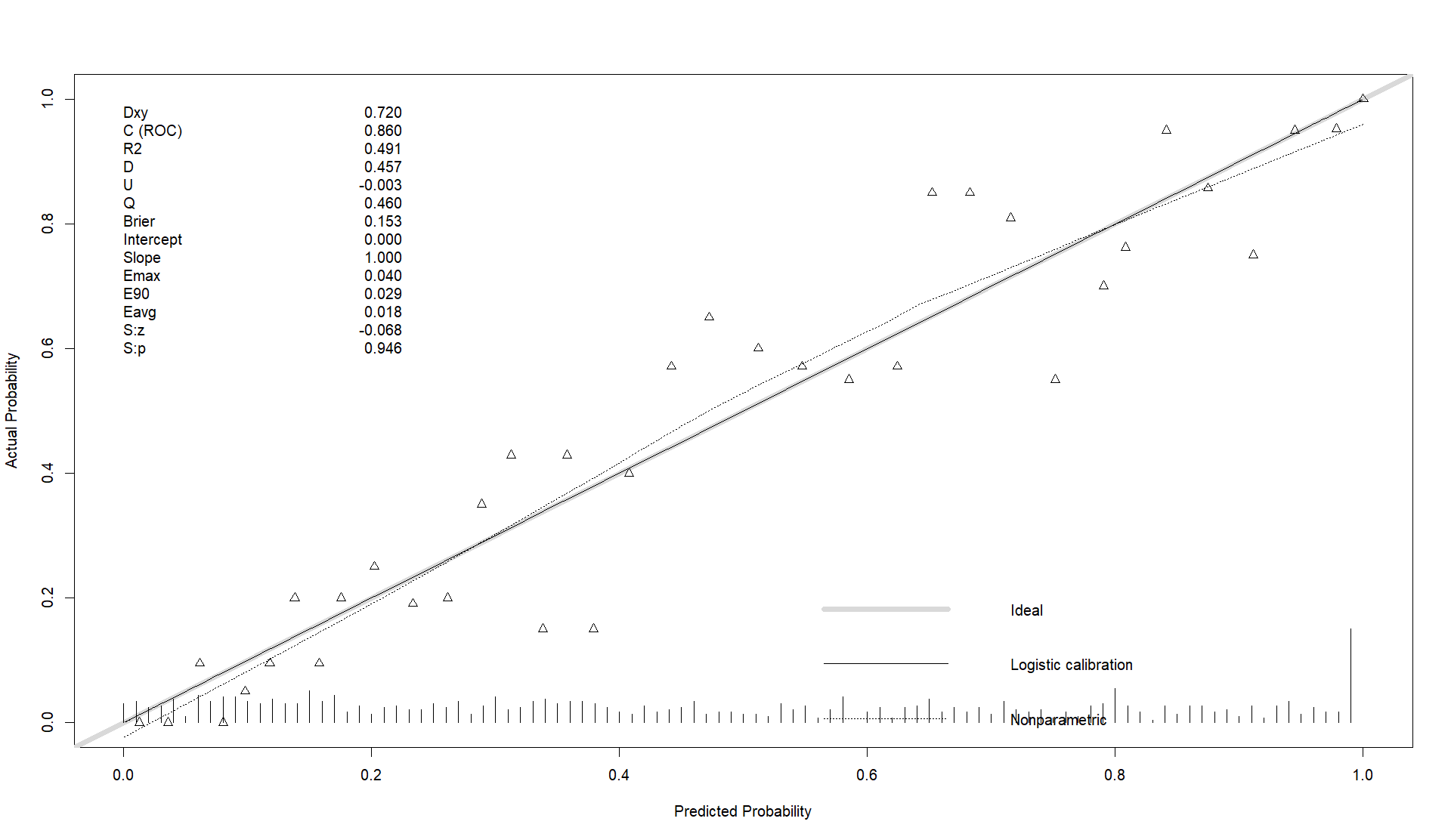


**Supplement figure 5. Calibration curves of the predictive EUGR risk nomogram in validation set.**


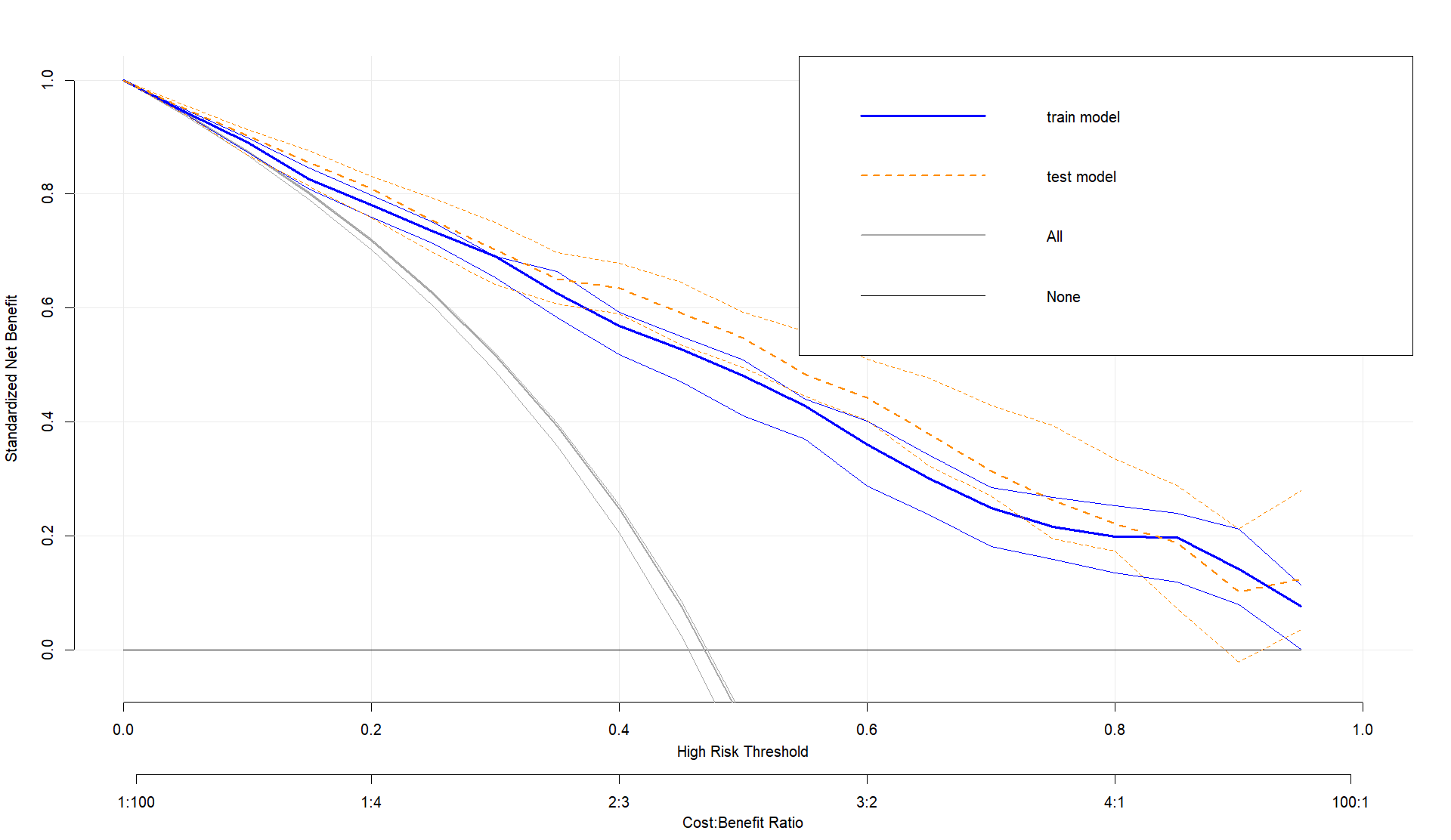


**Supplement figure 6.** **Decision curve analysis for the EUGR risk nomogram in the training set and validation set**
